# Identification of the optimal growth charts for use in a preterm population: An Australian state-wide retrospective cohort study

**DOI:** 10.1371/journal.pmed.1002923

**Published:** 2019-10-04

**Authors:** Natasha L. Pritchard, Richard J. Hiscock, Elizabeth Lockie, Michael Permezel, Monica F. G. McGauren, Amber L. Kennedy, Brittany Green, Susan P. Walker, Anthea C. Lindquist

**Affiliations:** 1 Department of Obstetrics and Gynaecology, University of Melbourne, Melbourne, Victoria, Australia; 2 Department of Obstetrics and Gynaecology, Mercy Hospital for Women, Heidelberg, Victoria, Australia; 3 Mercy Perinatal, Mercy Hospital for Women, Heidelberg, Victoria, Australia; University of Manchester, UNITED KINGDOM

## Abstract

**Background:**

Preterm infants are a group at high risk of having experienced placental insufficiency. It is unclear which growth charts perform best in identifying infants at increased risk of stillbirth and other adverse perinatal outcomes. We compared 2 birthweight charts (population centiles and INTERGROWTH-21st birthweight centiles) and 3 fetal growth charts (INTERGROWTH-21st fetal growth charts, World Health Organization fetal growth charts, and Gestation Related Optimal Weight [GROW] customised growth charts) to identify which chart performed best in identifying infants at increased risk of adverse perinatal outcome in a preterm population.

**Methods and findings:**

We conducted a retrospective cohort study of all preterm infants born at 24.0 to 36.9 weeks gestation in Victoria, Australia, from 2005 to 2015 (28,968 records available for analysis). All above growth charts were applied to the population. Proportions classified as <5th centile and <10th centile by each chart were compared, as were proportions of stillborn infants considered small for gestational age (SGA, <10th centile) by each chart. We then compared the relative performance of non-overlapping SGA cohorts by each chart to our low-risk reference population (infants born appropriate size for gestational age [>10th and <90th centile] by all intrauterine charts [AGA_all_]) for the following perinatal outcomes: stillbirth, perinatal mortality (stillbirth or neonatal death), Apgar <4 or <7 at 5 minutes, neonatal intensive care unit admissions, suspicion of poor fetal growth leading to expedited delivery, and cesarean section. All intrauterine charts classified a greater proportion of infants as <5th or <10th centile than birthweight charts. The magnitude of the difference between birthweight and fetal charts was greater at more preterm gestations. Of the fetal charts, GROW customised charts classified the greatest number of infants as SGA (22.3%) and the greatest number of stillborn infants as SGA (57%). INTERGROWTH classified almost no additional infants as SGA that were not already considered SGA on GROW or WHO charts; however, those infants classified as SGA by INTERGROWTH had the greatest risk of both stillbirth and total perinatal mortality. GROW customised charts classified a larger proportion of infants as SGA, and these infants were still at increased risk of mortality and adverse perinatal outcomes compared to the AGA_all_ population. Consistent with similar studies in this field, our study was limited in comparing growth charts by the degree of overlap, with many infants classified as SGA by multiple charts. We attempted to overcome this by examining and comparing sub-populations classified as SGA by only 1 growth chart.

**Conclusions:**

In this study, fetal charts classified greater proportions of preterm and stillborn infants as SGA, which more accurately reflected true fetal growth restriction. Of the intrauterine charts, INTERGROWTH classified the smallest number of preterm infants as SGA, although it identified a particularly high-risk cohort, and GROW customised charts classified the greatest number at increased risk of perinatal mortality.

## Abstract 2

Anthea Clare Lindquist and colleagues reveal the growth charts that are best used for identifying certain perinatal outcomes in preterm babies.

## Author summary

**Why was this study done?:**

- Preterm infants are a high-risk group for growth restriction resulting from placental dysfunction.
- Growth charts are widely used during antenatal ultrasound to identify fetuses at increased risk of adverse outcomes related to being small for gestational age (SGA), but these charts vary in their ability to identify the most at risk growth-restricted infants in utero.
- This study was performed to ascertain whether intrauterine or birthweight growth charts are most appropriate in a preterm population, in particular, which chart most accurately identifies the SGA infant at increased risk of adverse perinatal outcomes.

**What did the researchers do and find?:**

- We performed a state-wide retrospective cohort analysis that included data on 28,968 preterm births from Victoria, Australia, during the period 2005–2015.
- Using <10th centile as the cut-off for SGA, our analysis compared the sensitivity and specificity of 5 growth charts—2 birthweight charts and 3 intrauterine charts—for stillbirth and other adverse perinatal outcomes.
- Our study found that intrauterine charts classify a significantly greater proportion of the preterm population as SGA compared with birthweight charts at this gestation. Of the fetal charts, INTERGROWTH classified a smaller proportion as SGA, but this cohort had the greatest risk of perinatal mortality and morbidity.
- WHO and GROW charts classified an additional subgroup that was also at increased risk of perinatal mortality and morbidity, with GROW charts shown to be the most sensitive in the detection of SGA infants at increased risk of adverse perinatal outcomes.

**What do these findings mean?:**

- Our findings highlight the trade-off that exists between the greater specificity of INTERGROWTH fetal charts and the higher sensitivity of WHO and GROW growth charts.
- The differences between these growth charts are likely to be the result, at least in part, of differences in the development of the charts and the techniques and assumptions that underpin them.

## Introduction

Being born small for gestational age (SGA) is associated with greater risk of perinatal death, neonatal morbidity, adverse neurodevelopmental outcomes, and poor long-term adult health [1–3]. For the infant born both small and preterm, these risks are compounded [4]. Approximately 1 in every 9 infants is born preterm, and higher rates of indicated preterm births [5], as well as higher survival rates of those born extremely preterm [6], mean that it is becoming increasingly important to correctly identify preterm infants who are at greater risk of adverse outcomes as a result of being born SGA [7–10].

Unlike a term population, in which the majority of neonates are born healthy and appropriate size for gestational age (AGA), a preterm population contains a higher proportion of truly growth-restricted infants. This is thought to be due both to placental insufficiency triggering labour in some cases of spontaneous preterm birth [11,12] and to an increase in iatrogenic delivery because of prenatally identified fetal growth restriction (FGR) or preeclampsia [12].

Although it has been demonstrated that preterm newborns have significantly lower birthweights than would be expected from the estimated weights of fetuses that remain in utero and proceed to term [13], population birthweight centiles are commonly used, both clinically and in research, to define the SGA preterm infant. This approach runs the risk of systematically underestimating the occurrence of SGA and therefore under-diagnosing placental insufficiency causing pathological growth restriction in the preterm population. The implications of this are a lost opportunity to provide both increased antenatal surveillance and timely delivery that could reduce the risk of stillbirth [14], in addition to tailored postnatal care in the special care nursery and beyond in order to minimise the risk of adverse long-term outcomes.

Published data examining a combined preterm/term population has suggested that SGA screen-positive rates are higher for fetal growth standards than for birthweight standards [15]. In response to concerns about the utility of birthweight standards in appropriately identifying the SGA preterm infant, several alternative fetal charts have been proposed. These charts allow comparison of the birthweight of the preterm infant to that of the relatively healthy cohort of fetuses that remain in utero [16]. Two main types of fetal charts have been suggested: those derived from healthy populations with a low risk of fetal growth restriction, which therefore represent optimal fetal weight, and customised birthweight charts, where maternal characteristics such as height and weight are taken into consideration, in order to try to identify the ideal infant birthweight for a particular mother.

Two charts derived from ‘ideal’ populations of healthy weight mothers include the INTERGROWTH-21st and the World Health Organization (WHO) fetal charts. INTERGROWTH was a population-based project across 8 countries that assessed newborn size in mothers with adequate nutrition and antenatal care. INTERGROWTH published preterm birthweight charts for clinical use [17], as well as fetal growth charts derived from the same study. The WHO fetal charts were based on a smaller pregnant population, with similar inclusion criteria to INTERGROWTH, from 10 countries [18]. A commonly used customised chart is the Gestation Related Optimal Weight (GROW) charts, which have been demonstrated to more accurately classify pathologically SGA infants in a term population [19–22], with options to fully customise on maternal height, weight, parity, and ethnicity.

The first aim of our study was to compare 2 birthweight charts (population birthweight charts and the INTERGROWTH birthweight charts) with 3 intrauterine charts (INTERGROWTH fetal charts, WHO fetal charts, and GROW customised centiles) to ascertain whether intrauterine or birthweight centiles were most appropriate in a preterm population. Our second aim was to identify which fetal chart most accurately identified the SGA infant at increased risk of adverse perinatal outcomes associated with placental insufficiency.

## Methods

### Study population and data collection

A retrospective cohort study was conducted on all preterm infants who were delivered in Victoria, Australia, from 1 January 2005 to 31 December 2015. Data were obtained from the Consultative Council on Obstetric and Paediatric Mortality and Morbidity, which is the central agency that collects data on obstetric and perinatal outcomes within the state.

Prior to data cleaning and analysis, a plan was formulated to determine the inclusion and exclusion criteria, how gestational age would be defined in the case of stillbirth, how implausible data values would be managed in the analysis, and how the analysis would proceed, with a step-wise progression from examining all charts to comparison between a select few (S1 Text). Singleton pregnancies from 24.0 to 36.9 weeks gestation at delivery were included. Those less than 24 weeks’ gestation were excluded due to significant variation in resuscitation preferences and outcomes. Other exclusion criteria included multiple pregnancy, congenital anomaly, termination of pregnancy, missing or implausible birthweight, and missing fetal sex. Additionally, given the importance of gestation in determining a birthweight centile, those in whom gestation in days was not recorded, and those with any uncertainty regarding the exact gestation, were excluded.

Gestation in days was calculated based on the date of birth and the last normal menstrual period (pre-2010) or the date of birth and estimated due date (2010 onwards). Maternal height and weight data used for customised centiles were based on measurements recorded at the obstetric booking visit. Parity was defined as the number of previous births (live or stillborn) over 20 weeks gestation. Maternal age was recorded to the nearest year at booking, and birthweight was recorded in grams. Maternal indigenous status was classified based on self-identification as an Indigenous Australian. Ethnicity was not included as a variable in the analysis due to limitations of the dataset, which included data on self-identified country of birth only. Country of birth has been shown to be a poor surrogate for ethnicity in Australia, where the population is characterised by high rates of migration and interethnic marriage [23].

### Growth charts

Five separate growth charts were applied to our population: 2 birthweight charts (Australian population charts and INTERGROWTH), and 3 intrauterine charts (INTERGROWTH fetal growth charts, WHO fetal growth charts, and GROW customised charts). In order to identify SGA infants using intrauterine charts, intrauterine-derived growth centiles were applied to the birthweight of preterm infants included in our study. For all charts, <10th centile was considered SGA.

Population birthweight charts were those published by Dobbins et al. in 2012 [24], which are the most contemporary Australian population birthweight charts, and considered to be the standard amongst this population. These were based on 2.53 million singleton live births in Australia, between 1998 and 2007, which included 145,015 preterm infants.

The INTERGROWTH daily birthweight centiles for male and female infants from 24 to 36 completed weeks gestation were used [25]. Late preterm (33–37 weeks gestation) newborn birthweight data were derived from the INTERGROWTH primary cohort of infants with no congenital malformations born to healthy women from 8 countries aged 18–35 years, with a BMI between 18.5 and 30 kg/m^2^, living in proximity to antenatal care, and with no history of consecutive miscarriages, stillbirth, or neonatal death or other known risk factors for FGR [25]. The very preterm newborn standards (24–33 weeks gestation) were based on 408 neonates from the same overall cohort, but who were not strictly excluded if they had certain risk factors for FGR [26].

The estimated fetal weight calculation for the INTERGROWTH fetal growth charts was derived from 2,404 infants born within 14 days of their most recent ultrasound, with 130 (5.4%) born at less than 37 weeks, taken from both the INTERGROWTH-21st Fetal Growth Longitudinal Study (low-risk women) and the INTERBIO-21st Fetal Study (unselected cohort of pregnant women) [27]. This calculation was then applied to the healthy INTERGROWTH population described above in order to generate the in utero fetal centiles.

The WHO estimated fetal weight centiles were derived from 1,387 infants who had ultrasounds every 4 weeks until delivery. Similar to INTERGROWTH, participants were infants with no congenital anomalies from a cohort of healthy women from 10 countries aged 18–40 years, with a BMI between 18 and 30 kg/m^2^, living in proximity to antenatal care, and with no known risk factors for FGR [18]. Centiles in between ultrasounds were estimated using quantile regression and smoothed by polynomial functions of gestational age. Fetal sex was accounted for when WHO charts were applied to our study population.

GROW centiles were used for customised standards (version 8.0.3, 2018), validated previously by Gardosi and colleagues [21]. The GROW charts are derived from global population birthweight statistics. The charts apply to completed days of gestation, using coefficients derived from large population databases, to predict optimal fetal growth at term. Predicted weight at preterm gestations is determined by a fetal weight curve extrapolated backwards from term weights. GROW charts provide the option of customisation on multiple characteristics including maternal height, weight, parity, and ethnicity and fetal sex. For the purpose of this study, customisation was performed using fetal sex and maternal height, weight, and parity, but not ethnicity. Weight was coded as missing if <35 kg, height if <125 cm, and height and weight if BMI was <14 kg/m^2^ or not recorded. For the purpose of customisation, missing data defaulted to global population averages.

In order to assess the relative validity of the charts, the SGA population was calculated for each chart, in addition to ‘non-overlapping’ SGA populations, i.e., those classified as SGA by one definition but not by another.

We also assessed 2 additional cohorts: SGA_all_, which was defined as those infants classified as <10th centile by all intrauterine charts (INTERGROWTH, WHO, and GROW), and AGA_all_, which was all infants classified as >10th and <90th percentile on all intrauterine charts. SGA_all_ was used to identify a particularly high-risk cohort, considered growth restricted by all definitions, and AGA_all_ to identify a low-risk population, considered by no growth chart to be either inadequately or excessively grown. Comparisons were made between AGA_all_ and each of the populations and sub-populations defined as SGA.

### Outcomes

Our primary outcome was perinatal mortality, defined as either a stillborn fetus or a neonatal death within 28 days of delivery. Secondary outcomes included a 5-minute Apgar score (Ap^5^) of <7 or <4, or admission to the neonatal intensive care unit (NICU). Special care nursery admissions were not used as an outcome given the high likelihood of any preterm infant being admitted there. Suspicion of poor fetal growth as an indication for induction or expedited delivery was also noted. Caesarean section rate, both planned and unplanned (emergency), was also included as a secondary outcome.

Following precedent set by existing studies [28], birthweight centiles for stillbirths were examined in 2 ways—first, based on unadjusted gestational age at birth, and second, after adjusting for the likely time interval between fetal demise and delivery, to avoid overestimating the relative proportions of stillborn infants that were SGA. The adjusted gestation-specific weight centile included a correction of ‘death to delivery delay’ of 48 hours in each case.

### Statistical analysis

Baseline characteristics of the population were summarised by mean (standard deviation), median (25th–75th percentile), and number (%) according to type and distribution. For each SGA classification, outcomes are reported as the point estimate and Wilson 95% confidence interval both overall and for each gestational age-based stratum.

Re-classification of outcome due to application of the 5 SGA growth charts determines that the outcome proportions are clustered within infants, and this must be accounted for in the analysis. We used fixed effects conditional Poisson regression with robust variance estimates to provide relative risk ratios, 95% confidence intervals, and associated *p*-values between different SGA classification methods (growth charts). Gestational-age-based strata were incorporated into this regression model where appropriate. Significance level was 2-sided, set at 0.05, and not adjusted for multiple comparisons. Statistical analysis was conducted using GraphPad Prism version 7 and Stata version 14.

### Ethics

Ethics approval for the project was obtained from the Mercy Health Human Research Ethics Committee (approval project number R16-10). As this was a retrospective cohort study using de-identified data, individual patient consent was not required.

## Results

**Fig 1:**
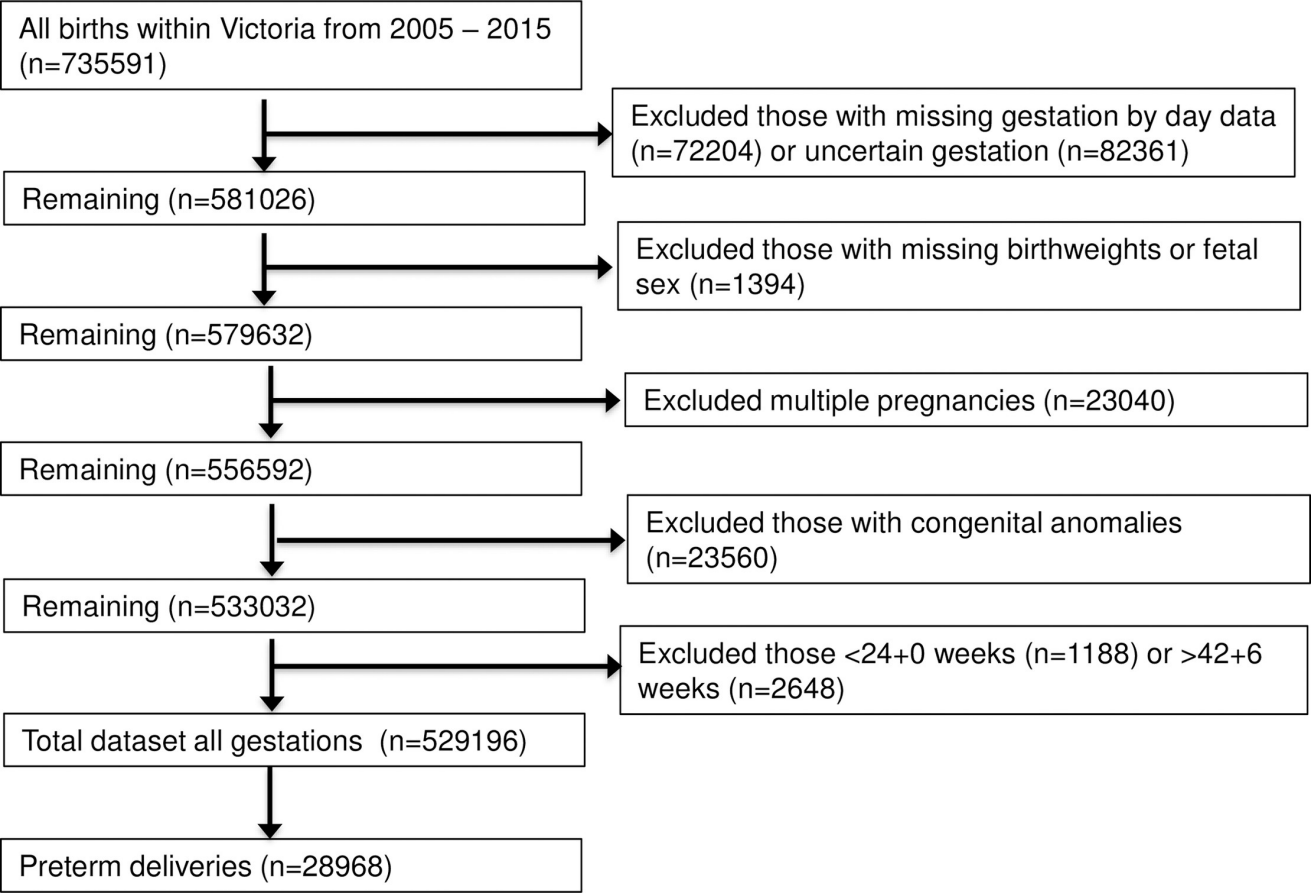
Flow diagram of exclusion criteria.

During the study timeframe, there were 735,591 births in Victoria, of which 206,395 were excluded because of multiple pregnancies, congenital anomalies, termination of pregnancy, gestation less than 24.0 weeks, missing or implausible birthweights, or missing fetal sex. Given the importance of gestation in determining birthweight centiles, those in whom gestation in days was not recorded and those with any uncertainty regarding the exact gestation were excluded (Fig 1). Of the 529,196 births remaining for analysis, there were 28,968 (5.5%) preterm deliveries between 24.0 to 36.9 weeks gestation included in this study.

### Proportion classified as SGA by different growth standards

We examined the proportions of infants classified as <5th and <10th centile by all 5 growth charts (Table 1). Both the Australian population and INTERGROWTH birthweight charts classified the expected proportion of infants as <5th and <10th centile overall. All 3 intrauterine charts classified greater proportions of infants as <10th centile than the birthweight charts, and this difference was highly significant (*p* < 0.001) for all comparisons across the total preterm population.

**Table 1:** Proportion of infants classified as <5th and <10th centile by different growth charts.

Classification	Birthweight charts		Intrauterine charts		
	Population centiles	INTERGROWTH birthweight charts	INTERGROWTH fetal charts	WHO fetal charts	GROW customised charts
**<5th centile**					
Overall (*n* = 28,969)	1,614 (5.6) (5.3–5.8)	1,825 (6.3) (6.0–6.6)	2,704 (9.3) (9.0–9.7)	4,007 (13.8) (13.4–14.2)	4,601 (15.9) (15.5–16.3)
<28 weeks (*n* = 1,155)	155 (13.4) (11.6–15.5)	189 (16.4) (14.3–18.6)	401 (34.7) (32.0–37.5)	355 (30.7) (28.1–33.5)	366 (31.7) (29.1–34.4)
28–32 weeks (*n* = 2,346)	167 (7.1) (6.1–8.2)	237 (10.1) (8.9–11.4)	514 (21.9) (20.3–23.6)	652 (27.8) (26.0–29.6)	713 (30.4) (28.6–32.3)
>32 weeks (*n* = 25,467)	1,292 (5.1) (4.8–5.3)	1,399 (5.5) (5.2–5.8)	1,789 (7.0) (6.7–7.3)	3,000 (11.8) (11.4–12.2)	3,522 (13.8) (13.4–14.3)
**<10th centile**					
Overall (*n* = 28,969)	3,103 (10.7) (10.4–11.1)	3,144 (10.9) (10.5–11.2)	3,876 (13.4) (13.0–13.8)	5,679 (19.6) (19.1–20.1)	6,463 (22.3) (21.8–22.8)
<28 weeks (*n* = 1,155)	228 (19.7) (17.5–22.1)	248 (21.5) (19.2–23.9)	455 (39.4) (36.6–42.2)	410 (35.5) (32.8–38.3)	443 (38.4) (36.6–41.2)
28–32 weeks (*n* = 2,346)	294 (12.5) (11.3–13.9)	340 (14.5) (13.1–16.0)	623 (26.6) (24.8–28.4)	789 (33.6) (31.7–35.6)	898 (38.3) (35.6–40.3)
>32 weeks (*n* = 25,467)	2,581 (10.1) (9.8–10.5)	2,556 (10.1) (9.7–10.4)	2,798 (11.0) (10.6–11.4)	4,480 (17.6) (17.1–18.1)	5,122 (20.1) (19.6–20.6)

Data presented as number (%) (95% CI of percent).

The INTERGROWTH fetal charts identified a higher number of SGA infants than the INTERGROWTH birthweight charts, despite being derived from the same population (*p <* 0.001) (S1 Table). The magnitude of this difference was greater at the more preterm gestations, with a relative risk ratio (INTERGROWTH fetal charts compared to INTERGROWTH birthweight charts for <10th centile) of 1.83 (95% CI 1.69–2.00) at <28 weeks gestation compared with 1.09 (95% CI 1.08–1.11) at >32 weeks gestation (S1 Table).

**Fig 2:**
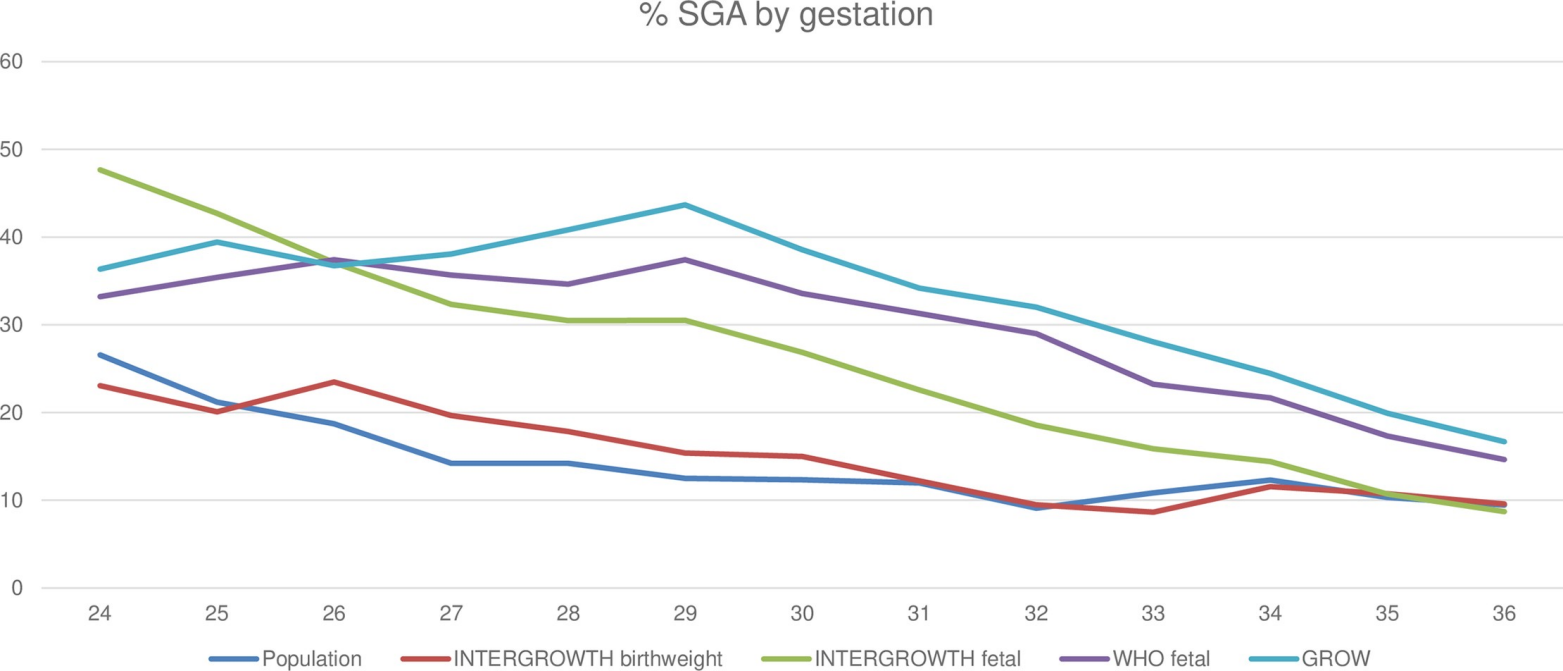
Proportion classified as SGA by each chart by week of gestation. GROW, Gestation Related Optimal Weight; SGA, small for gestational age.

Similar findings occurred across all intrauterine charts, with the greatest difference in the proportion of infants classified as SGA seen in more preterm cohorts, and the discrepancy narrowing closer to term (Fig 2). All 5 growth charts, however, classified a greater proportion of infants as <10th centile at <28 weeks than at >32 weeks (*p <* 0.001).

### Proportion of all stillbirths classified as SGA by different growth charts

Overall, GROW customised charts classified the greatest number of preterm infants as SGA (22.3% overall for <10th centile), and this was consistent across all gestations (38.4% at <28 weeks, 38.3% at 28–32 weeks, and 20.1% at >32 weeks) (Table 1).

**Fig 3:**
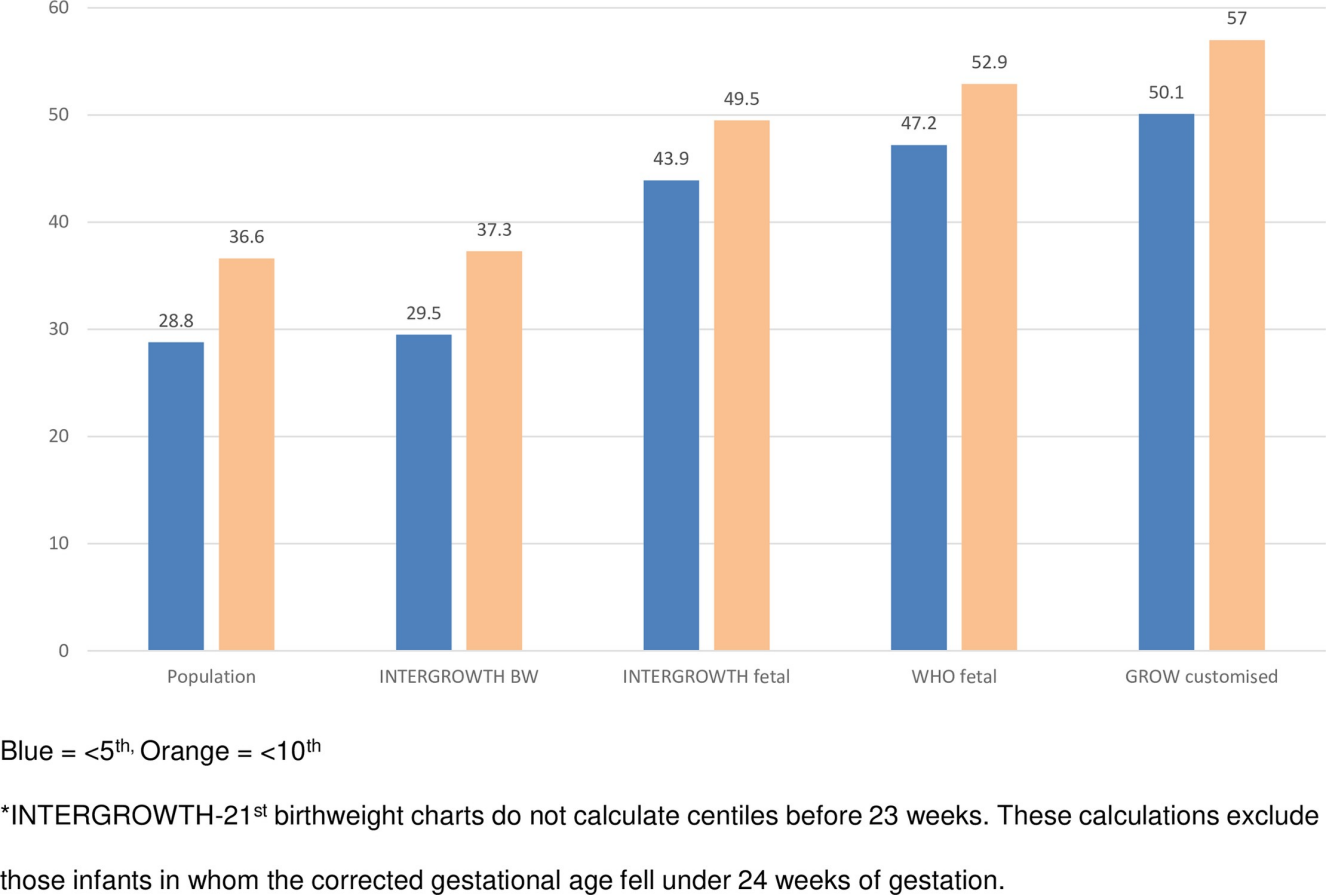
Proportion of all preterm stillborn infants (*n* = 865) classified as <5th or <10th centile by each growth chart. BW, birthweight; GROW, Gestation Related Optimal Weight.

All 3 intrauterine charts classified a greater proportion of stillborn infants as <5th or <10th centile compared to the birthweight charts (*p <* 0.001 for all comparisons) (Fig 3). This remained significant when adjusted for gestational age at demise. GROW customised charts classified the greatest proportion as SGA, with over half classified as SGA, in both analyses unadjusted and adjusted for gestational age at death.

### Comparison of intrauterine growth charts: INTERGROWTH, WHO, and GROW

**Fig 4:**
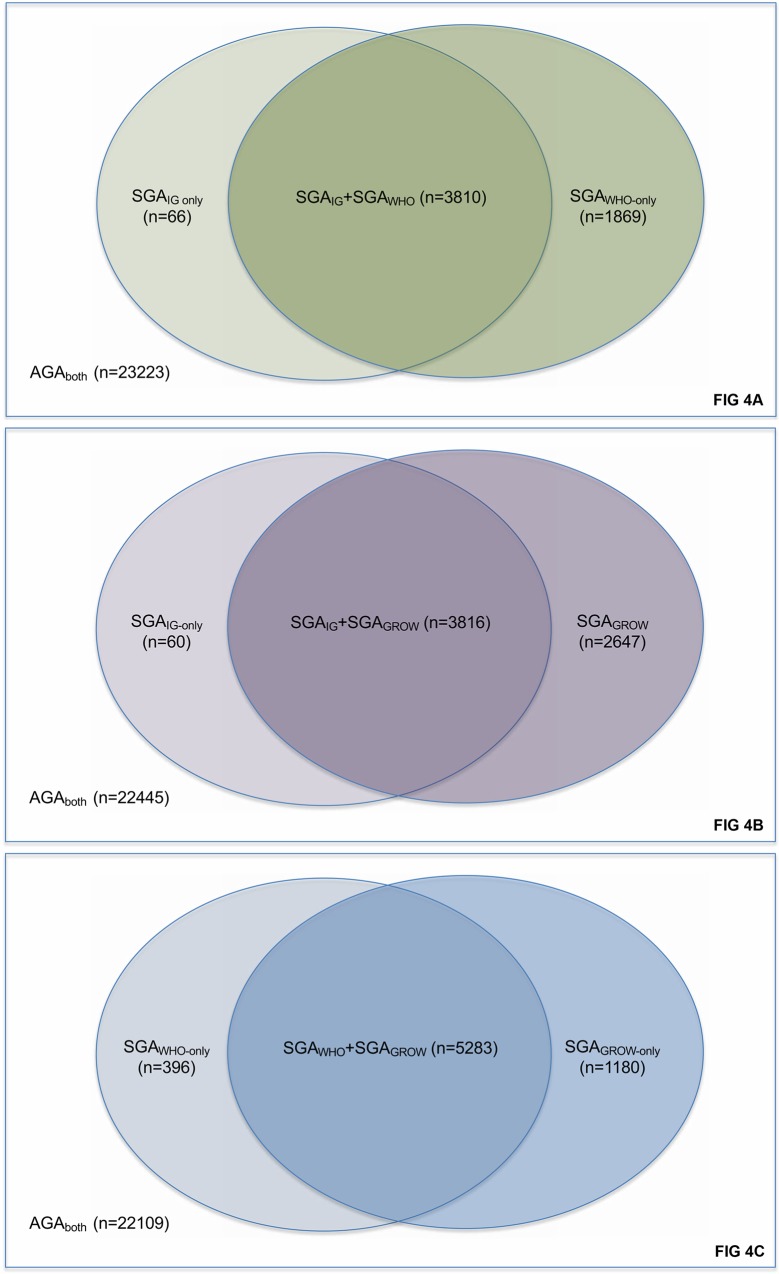
Comparison of intrauterine growth charts. (A) Comparison of populations classified as SGA by INTERGROWTH and WHO fetal charts. (B) Comparison of populations classified as SGA by INTERGROWTH fetal charts and GROW customised charts. (C) Comparison of populations classified as SGA by WHO fetal charts and GROW customised charts. AGA, appropriate size for gestational age; GROW, Gestation Related Optimal Weight; IG, INTERGROWTH; SGA, small for gestational age.

The INTERGROWTH fetal charts classified only 66 infants (0.23%) as SGA that were not also classified as SGA by the WHO charts, whereas 1,869 infants (6.5%) were classified as SGA by WHO but not INTERGROWTH (Fig 4A). The INTERGROWTH fetal charts classified only 60 infants (0.21%) as SGA that were not classified by as SGA by GROW charts, whereas 2,647 infants (9.1%) were classified as SGA by GROW but not INTERGROWTH (Fig 4B). When comparing WHO charts with GROW charts, 396 (1.4%) were considered SGA only by WHO, and 1,180 (4.1%) as SGA only by GROW, with 5,283 (18.2%) considered SGA by both charts (Fig 4C).

Baseline characteristics of SGA populations based on the 3 intrauterine growth charts are show in Table 2. Of note, fewer infants whose mothers were nulliparous were classified as SGA by GROW customised charts (49.1%) than by the INTERGROWTH (56.5%) or WHO (55.4%) charts. Fewer male infants were classified by the INTERGROWTH fetal charts as SGA (43.7%) than by WHO (51.3%) or GROW (51.5%) charts. Those classified as SGA by INTERGROWTH fetal charts also had, on average, slightly earlier gestation at delivery (242 days) than those classified by WHO (245 days) or GROW (245 days).

**Table 2:** Baseline maternal and infant characteristics for infants classified as AGA_all_ and SGA by fetal growth charts (unadjusted data presented).

Characteristic	AGA_all_ (*n* = 16,470)	SGA_all_ (*n* = 3,786)	SGA_IG_ (*n* = 3,876)	SGA_WHO_ (*n* = 5,679)	SGA_GROW_ (*n* = 6,463)
**Maternal characteristics**					
Age, mean (SD)	31.0 (5.7)	31.1 (16.8)	31.0 (16.7)	30.9 (14.1)	31.3 (18.0)
Height, mean (SD)	163 (15.9)	162 (7.1)	162 (7.1)	162 (7.2)	163 (7.1)
BMI, mean (SD)	25.5 (6.0)	25.7 (6.7)	25.7 (6.7)	25.5 (6.5)	26.4 (6.7)
Indigenous, *n* (%)	227 (1.4)	89 (2.4)	91 (2.3)	126 (2.2)	152 (2.4)
Nulliparous, *n* (%)	8,203 (49.8)	2,133 (56.3)	2,190 (56.5)	3,145 (55.4)	3,172 (49.1)
**Infant characteristics**					
Birthweight, median (IQR)	2,570 (2,289.3–2,790)	1,675 (1,198–1,965)	1,665 (1,166–1,962)	1,830 (1,400–2,110)	1,870 (1,440–2,150)
Gestation at delivery, median (IQR)	251 (241–255)	242 (221–253)	242 (220–253)	245 (227–253)	245 (228–254)
Male, *n* (%)	8,940 (54.3)	1,679 (44.3)	1,694 (43.7)	2,914 (51.3)	3,326 (51.5)

AGA_all_, appropriate size for gestational age (>10th and <90th centile) by all intrauterine charts; SGA_all_, small for gestational age (<10th centile) by all intrauterine charts; SGA_IG_, small for gestational age (<10th centile) by INTERGROWTH fetal charts; SGA_WHO_, small for gestational age (<10th centile) by WHO fetal charts; SGA_GROW_, small for gestational age (<10th centile) by GROW customised charts.

**Fig 5:**
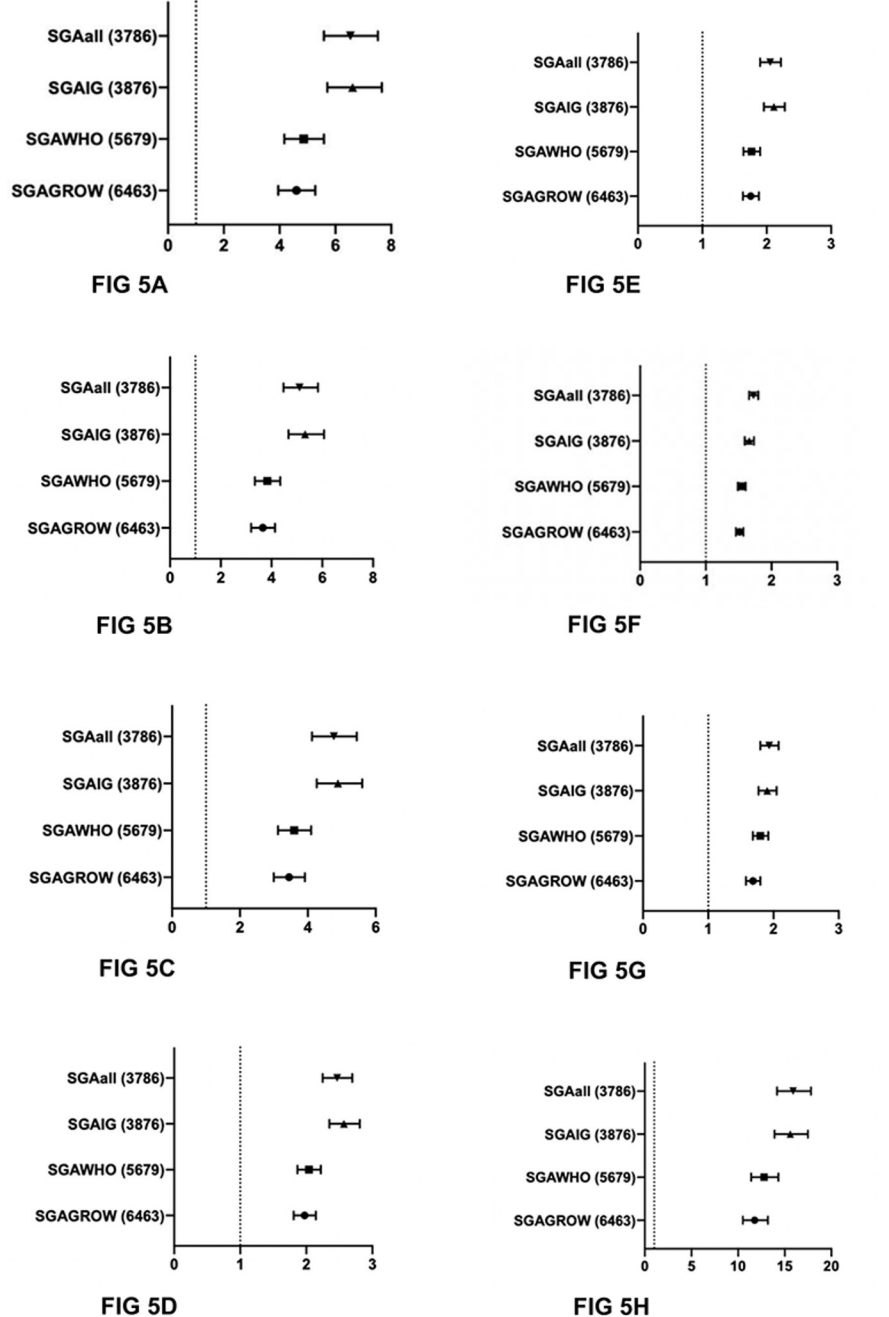
Relative risk ratios of <10th centile populations for adverse outcomes by fetal charts INTERGROWTH, WHO, and GROW, compared with the AGA_all_ population. (A) Stillbirth. (B) Perinatal mortality (stillbirth or neonatal death). (C) Apgar < 4 at 5 minutes. (D) Apgar < 7 at 5 minutes. (E) Neonatal intensive care unit admission. (F) Unplanned (emergency) cesarean section rate. (G) Fetal distress indication for operative delivery. (H) Suspected poor fetal growth indication for induction or operative birth. AGA_all_, appropriate size for gestational age by all intrauterine charts; GROW, Gestation Related Optimal Weight; SGAall, small for gestational age by all intrauterine charts; SGAIG, small for gestational age by INTERGROWTH fetal charts; SGAWHO, small for gestational age by WHO fetal charts; SGAGROW, small for gestational age by GROW customised centiles.

Comparison of the primary and secondary outcomes between the SGA populations and AGA_all_ is shown in Fig 5. Comparisons with AGA_all_ were made for SGA_all_, SGA by INTERGROWTH fetal charts (SGA_IG_), SGA by WHO fetal charts (SGA_WHO_), and SGA by GROW charts (SGA_GROW_). There was little difference in the populations SGA_IG_ and SGA_all_, given that INTERGROWTH classified the smallest number of infants as SGA. As such, the relative risk ratios for those 2 populations are very similar across all outcomes. Infants classified as SGA_IG_ had consistently higher risk of all adverse outcomes.

The populations not classified as SGA by INTERGROWTH charts but classified as SGA by WHO charts (SGA_WHO_AGA_IG_) or by GROW charts (SGA_GROW_AGA_IG_) were also compared to the AGA_all_ population. Compared to the AGA_all_ population, infants in the SGA_WHO_AGA_IG_ cohort were at significantly increased risk of stillbirth, admission to the NICU, and cesarean section delivery, but not perinatal mortality or low Apgar (S2 Table). Infants in the SGA_GROW_AGA_IG_ cohort were at increased risk of perinatal mortality, Ap^5^ < 4, Ap^5^ < 7, admission to the NICU, and delivery by cesarean section. The subgroups classified as SGA by INTERGROWTH but not by WHO or GROW charts were not examined, given the lack of power associated with such small numbers.

### WHO intrauterine charts compared with GROW customised charts

Table 3 presents the maternal and infant characteristics and perinatal outcomes of the non-overlapping SGA populations from the WHO and GROW charts. The median gestation at delivery and birthweight were comparable, but SGA_WHO_AGA_GROW_ had a higher proportion of nulliparous women (79.4% versus 29.1%, *p <* 0.001), as well as a significantly lower average BMI (21.7 versus 28.6 kg/m^2^, *p <* 0.001), significantly shorter average height (156 versus 166 cm, *p <* 0.001), and significantly younger average age (29.7 versus 31.6 years, *p <* 0.001) than the group SGA_GROW_AGA_WHO_.

**Table 3:** Baseline characteristics and perinatal outcomes for non-overlapping populations classified as small for gestational age by WHO fetal charts or GROW charts.

Characteristic or outcome	AGA_all_ (*n* = 16,470)	SGA_WHO_AGA_GROW_ (*n* = 396)	SGA_GROW_AGA_WHO_ (*n* = 1,180)
***Baseline characteristics***			
**Maternal**			
Age, mean (SD)	31.0 (5.7)	29.7 (5.5)	31.6 (5.8)
Height, mean (SD)	163 (15.9)	156 (6.4)	166 (6.5)
BMI, mean (SD)	25.5 (6.0)	21.7 (3.6)	28.6 (6.7)
Indigenous, *n* (%)	227 (1.4)	3 (0.8)	29 (2.5)
Nulliparous, *n* (%)	8,203 (49.8)	316 (79.4)	343 (29.1)
**Infant**			
Birthweight, median (IQR)	2,570 (2,289.3–2,790)	2,184 (1,924–2,300)	2,230 (1,866–2,410)
Gestation at delivery, median (IQR)	251 (241–255)	250 (348–253)	249 (235–256)
Male, *n* (%)	8,940 (54.3)	214 (53.8)	626 (53.1)
***Obstetric and perinatal outcomes***			
**Perinatal mortality**			
Total	375 (2.3)	12 (3.0)	54 (4.6)
	Ref	1.33 (0.76–2.34, *p* = 0.32)	2.00 (1.52–2.66, *p* < 0.001)
Stillbirth	275 (1.7)	9 (2.3)	44 (3.7)
	Ref	1.36 (0.71–2.62, *p* = 0.36)	2.23 (1.63–3.05, *p* < 0.001)
Neonatal death	100 (0.61)	3 (0.76)	10 (0.84)
	Ref	1.25 (0.40–3.92, *p* = 0.70)	1.40 (0.73–2.66, *p* = 0.31)
**Apgar score**			
Ap^5^ < 4	360 (2.2)	10 (2.5)	50 (4.2)
	Ref	1.16 (0.62–2.15, *p* = 0.65)	1.94 (1.45–2.59, *p* < 0.001)
Ap^5^ < 7	1,105 (6.7)	37 (9.3)	116 (9.8)
	Ref	1.39 (1.02–1.90, *p* = 0.04)	1.47 (1.22–1.76, *p* < 0.001)
**NICU admission**	1,618 (9.8)	46 (11.6)	174 (14.7)
	Ref	1.18 (0.90–1.56, *p* = 0.24)	1.50 (1.30–1.73, *p* < 0.001)
**Suspicion of poor growth**			
Operative delivery for fetal distress	1,901 (11.5)	81 (20.5)	159 (13.5)
	Ref	1.77 (1.45–2.16, *p* < 0.001)	1.17 (1.00–1.36, *p* = 0.05)
Induction or operative birth for suspected poor fetal growth	342 (2.1)	41 (10.4)	115 (9.7)
	Ref	4.99 (3.66–6.79, *p* < 0.001)	4.69 (3.83–5.75, *p* < 0.001)
**Cesarean section**			
Total	6,465 (39.3)	188 (47.5)	583 (49.4)
		1.21 (1.09–1.34, *p* < 0.001)	1.26 (1.18–1.34, *p* < 0.001)
Planned	Ref	58 (14.6)	176 (14.9)
	1,874 (11.4)	1.29 (1.01–1.64, *p* = 0.04)	1.31 (1.14–1.51, *p* < 0.001
Emergency	Ref	130 (32.8)	407 (34.5)
	4,591 (27.9)	1.18 (1.02–1.36, *p* = 0.03)	1.24 (1.14–1.34, *p* < 0.001)

Outcome data presented as number (%) and relative risk ratio (95% CI, *p*-value).

AGA_all_, appropriate size for gestational age (>10th centile and <90th centile) by all intrauterine charts; Ap^5^, 5-minute Apgar score; GROW, Gestation Related Optimal Weight; NICU, neonatal intensive care unit; SGA_WHO_AGA_GROW_, small for gestational age (<10th centile) by WHO fetal charts but appropriate size for gestational age (>10th centile) by GROW customised charts; SGA_GROW_AGA_WHO_, small for gestational age (<10th centile) by GROW customised charts but appropriate size for gestational age (>10th centile) by WHO fetal charts.

Compared to the healthy reference group of AGA_all_, SGA_GROW_AGA_WHO_ was at significantly increased risk of stillbirth and total perinatal mortality. However, there was no significant difference between SGA_WHO_AGA_GROW_ and AGA_all_ in regards to mortality. SGA_GROW_AGA_WHO_ was also at increased risk of Ap^5^ < 4 and Ap^5^ < 7, admission to the NICU, and delivery by planned or unplanned cesarean section. SGA_WHO_AGA_GROW_ was at increased risk of Ap^5^ < 7 and delivery by planned or unplanned cesarean section.

## Discussion

### Summary of findings

We performed a large, population-based analysis on 28,968 singleton preterm infants born in a single state in Australia at 24.0 to 36.9 weeks gestation, and compared the utility of 5 different growth charts in predicting risk of perinatal mortality and morbidity. The growth standards assessed included 2 birthweight charts (Australian population charts and INTERGROWTH charts) and 3 intrauterine charts (INTERGROWTH fetal charts, WHO fetal charts, and GROW customised charts). In keeping with existing literature [29], we confirmed that all 5 charts classify a greater proportion of infants as SGA at more preterm gestations (<28 weeks compared to >32 weeks) and that intrauterine charts classify a greater proportion of preterm infants as SGA than birthweight charts do. Preterm populations are known to be at greater risk of SGA, because of both iatrogenic delivery for placental insufficiency and the association between FGR and spontaneous preterm birth. As a result, fetal charts appear to be more representative of the true distribution of birthweights in a preterm population. Additionally, intrauterine charts classified greater proportions of stillborn infants as <5th or <10th centile, even after adjustment for gestational age at death. This has implications for clinical practice due to the association between placental insufficiency and stillbirth [30], particularly in the preterm period.

Of the fetal charts, infants classified as SGA by INTERGROWTH had the highest risk of adverse outcomes, but those that were SGA by GROW and WHO charts but AGA by INTERGROWTH still had an increased risk of adverse perinatal outcomes. Our findings highlight the trade-off that exists between the greater specificity of INTERGROWTH fetal charts and the higher sensitivity of WHO and GROW. This is likely to be the result of differences, at least in part, in the development of each growth chart and the techniques and assumptions that underpin them. For example, INTERGROWTH aimed to develop internationally valid aspirational targets for fetal growth by using optimised, ‘healthy’ populations. It is clear that in high-income countries, the INTERGROWTH growth curve remains left shifted, and thus the proportion of babies identified as being small in populations like ours will be reduced. INTERGROWTH will therefore have stronger associations with adverse outcome, but will have reduced sensitivity for detecting infants at risk. This was reflected by our study findings, which showed that, of the intrauterine charts, INTERGROWTH classified the smallest proportion of infants as SGA, yet this group had the highest risk of perinatal mortality and morbidity.

In contrast, the GROW fetal growth charts were developed using HADLOCK fetal calculations for term-optimised birthweights extrapolated backwards to determine preterm fetal standards, on which constitutional influences on fetal size are superimposed. This may explain why, as our study demonstrates, a larger proportion of infants are classified as SGA by GROW charts. Our study demonstrated that these infants are still at increased risk of mortality and adverse perinatal outcomes compared to the AGA_all_ population.

Given the similarities in their study design, it is surprising to find such substantial differences between the INTERGROWTH and WHO fetal charts. There are some differences in the respective populations, which may have had an impact on the findings; however, differences in the way in which the centile charts were derived from each study are likely to have had the greatest impact.

Comparison between the WHO and GROW charts, specifically the non-overlapping populations classified as SGA by only one or the other of these 2 charts, revealed that GROW classified a greater proportion of infants as SGA. Those classified as SGA by GROW charts only were at increased risk of stillbirth compared to the reference population (AGA_all_), but those classified as SGA by WHO charts only were not. This suggests a benefit in using customisation for maternal characteristics. Mothers of infants classified as SGA exclusively by GROW charts were taller, heavier, and more likely to be multiparous. It may be that customisation for SGA is of greater benefit in taller or heavier women, in whom a fetus may be expected to have a birthweight above the average. This group of infants is particularly important to identify, as maternal obesity can obscure clinical identification of the SGA fetus and is associated with a higher risk of stillbirth [31,32].

### Strengths and limitations

Our study contributes to the existing literature on the application of growth charts in a preterm population. It compared birthweight and intrauterine charts, and in doing so was able to demonstrate the benefits and limitations of each in a preterm population. Our data are comprehensive, covering an entire state over a decade, and give a contemporary representation of Australian obstetric practice.

We had a very large preterm sample size, which allowed rare but important outcomes such as stillbirth to be assessed. Other outcomes in a preterm population, such as low Apgar scores and NICU admissions, can be more difficult to link to placental insufficiency, as extreme preterm birth is itself associated with a greater chance of need for advanced resuscitation.

A significant challenge when comparing growth charts is the degree of overlap, with most infants classified as SGA on multiple charts, thus diminishing the power of comparison between groups. We were able to overcome this by examining sub-populations identified by only 1 growth chart, in order to assess the true difference between them. When women had missing data for height and weight, population defaults were used in customised growth charts, which would have likely underestimated the difference between customised and uncustomised GROW charts.

In our GROW customisation model, we did not customise on ethnicity. ‘Country of birth’ data were available for most women; however, extrapolating this to ethnicity can be unreliable, particularly amongst the Australian multiethnic population [23]. Additionally, it can be difficult to delineate the exact impact of ethnicity on birthweight, independent from the impact of socioeconomic status. We did customise for parity, as we felt that this was important, but this may have artificially normalised the lower birthweights of primiparous women, who are actually at increased risk of stillbirth [30].

Although <3rd centile and <10th centile are more commonly used cut-offs in clinical practice, published WHO fetal charts do not include <3rd centile standards, and so <5th centile was used in order to effectively compare all fetal charts.

### Conclusion

Intrauterine charts classify a significantly greater proportion of the preterm population as SGA compared with birthweight charts at this gestation. The fetal growth charts assessed in our study were each developed using different population assumptions and different techniques, and this likely explains, at least in part, the difference in sensitivity and specificity between charts. Of the fetal charts, INTERGROWTH classified a smaller proportion as SGA, but this cohort had the greatest risk of perinatal mortality and morbidity. WHO and GROW charts classified an additional subgroup that was also at increased risk of perinatal mortality and morbidity and would be missed if INTERGROWTH fetal charts alone were used. Additionally, compared to WHO charts, GROW identified an SGA subgroup at increased risk of perinatal mortality. Customised intrauterine charts appear to be the most sensitive in the detection of SGA infants at increased risk of adverse perinatal outcomes.

## Supporting information

S1 STROBE Checklist (PDF)

S1 Table Relative risk ratios and 95% confidence limits for infants classified as <5th and <10th centile by different growth charts. (DOCX)

S2 Table Baseline characteristics and perinatal outcomes for populations classified by WHO or GROW centiles, but not by INTERGROWTH, as SGA, in comparison to AGA_all_. (DOCX)

S1 Text Data analysis plan. (DOCX)

## Abbreviations

AGA: appropriate size for gestational age
Ap^5^: 5-minute Apgar score
FGR: fetal growth restriction
GROW: Gestation Related Optimal Weight
NICU: neonatal intensive care unit
SGA: small for gestational age
WHO: World Health Organization
