# Supplementary material for: Identification of the optimal growth charts for use in a preterm population: An Australian state-wide retrospective cohort study
Source: PLoS Med. 2019 Oct 4;16(10):e1002923. doi: 10.1371/journal.pmed.1002923 (PMC6777749; doi:10.1371/journal.pmed.1002923)
Supplement: S1 Table — (DOCX) [file pmed.1002923.s002.docx]

**S1 Table: Relative risk and 95% confidence limits for infants classified as <5^th^ and <10^th^ centile by different growth standards.**

| **<5^th^ CENTILE BIRTHWEIGHT STANDARDS** | | | |
| --- | --- | --- | --- |
|  | **INTERGROWTH-21st fetal chart** | **WHO fetal chart** | **GROW customised**  **chart** |
| **Population centiles (%)** | | | |
| **Overall (n=28969)** | 1.67  (1.62 – 1.73, p < 0.001) | 2.48  (2.39 – 2.58, p < 0.001) | 2.85  (2.74 – 2.96, p < 0.001) |
| **<28 weeks (n=1155)** | 2.59  (2.29 – 2.93, p < 0.001) | 2.29  (2.04 – 2.58, p < 0.001) | 2.36  (2.10 – 2.66, p < 0.001) |
| **28-32 weeks (n=2346)** | 3.08  (2.72 – 3.49, p < 0.001) | 3.90  (3.43 – 4.45, p < 0.001) | 4.27  (3.74 – 4.88, p < 0.001) |
| **>32 weeks (n=25467)** | 1.38  (1.34 – 1.43, p < 0.001) | 2.32  (2.23 – 2.42, p < 0.001) | 2.73  (2.61 – 2.85, p < 0.001) |
| **INTERGROWTH-21^st^ birthweight** | | | |
| **Overall (n=28969)** | 1.48  (1.44 – 1.53, p < 0.001) | 2.20  (2.12 – 2.27, p < 0.001) | 2.52  (2.43 – 2.61, p < 0.001) |
| **<28 weeks (n=1155)** | 2.12  (1.91 – 2.35, p < 0.001) | 1.88  (1.70 – 2.07, p < 0.001) | 1.94  (1.75 – 2.14, p < 0.001) |
| **28-32 weeks (n=2346)** | 2.17  (1.98 – 2.38, p < 0.001) | 2.75  (2.49 – 3.05, p < 0.001) | 3.01  (2.71 – 3.34, p < 0.001) |
| **>32 weeks (n=25467)** | 1.29  (1.25 – 1.31, p < 0.001) | 2.14  (2.06 – 2.23, p < 0.001) | 2.52  (2.42 – 2.61, p < 0.001) |
| **<10^th^ CENTILE BIRTHWEIGHT STANDARDS** | | | |
|  | **INTERGROWTH-21st fetal chart** | **WHO fetal chart** | **GROW customised**  **chart** |
| **Population centiles (%)** | | | |
| **Overall (n=28969)** | 1.25  (1.22 – 1.27, p < 0.001) | 1.83  (1.79 – 1.87, p < 0.001) | 2.08  (2.03 – 2.14, p < 0.001) |
| **<28 weeks (n=1155)** | 2.00  (1.82 – 2.19, p < 0.001) | 1.80  (1.65 – 1.96, p < 0.001) | 1.94  (1.78 – 2.13, p < 0.001) |
| **28-32 weeks (n=2346)** | 2.11  (1.95 – 2.30, p < 0.001) | 2.68  (2.45 – 2.94, p < 0.001) | 3.05  (2.78 – 3.35, p < 0.001) |
| **>32 weeks (n=25467)** | 1.08  (1.06 – 1.10, p < 0.001) | 1.74  (1.69 – 1.78, p < 0.001) | 1.98  (1.93 – 2.04, p < 0.001) |
| **INTERGROWTH-21^st^ birthweight** | | | |
| **Overall (n=28969)** | 1.23  (1.21 – 1.25, p < 0.001) | 1.81  (1.76 – 1.85, p < 0.001) | 2.06  (2.00 – 2.11, p < 0.001) |
| **<28 weeks (n=1155)** | 1.83  (1.69 – 2.00, p < 0.001) | 1.65  (1.53 – 1.79, p < 0.001) | 1.79  (1.64 – 1.94, p < 0.001) |
| **28-32 weeks (n=2346)** | 1.83  (1.71 – 1.97, p < 0.001) | 2.32  (2.14 – 2.51, p < 0.001) | 2.64  (2.43 – 2.87, p < 0.001) |
| **>32 weeks (n=25467)** | 1.09  (1.08 – 1.11, p < 0.001) | 1.75  (1.71– 1.80, p < 0.001) | 2.00  (1.95 – 2.06, p < 0.001) |

Presented as Relative Risk ratios (95%CI, p-value)
