# Supplementary material for: Identification of the optimal growth charts for use in a preterm population: An Australian state-wide retrospective cohort study
Source: PLoS Med. 2019 Oct 4;16(10):e1002923. doi: 10.1371/journal.pmed.1002923 (PMC6777749; doi:10.1371/journal.pmed.1002923)
Supplement: S2 Table — (DOCX) [file pmed.1002923.s003.docx]

**S2 Table: Baseline characteristics and perinatal outcomes for populations classified by WHO or GROW centiles as SGA, but not by INTERGROWTH, in comparison to AGA_all_**

|  | **AGA_all_ (n=16470)** | **SGA_WHO_AGA_IG_ (n=1869)** | **SGA_GROW_AGA_IG_ (n=2647)** |
| --- | --- | --- | --- |
| **BASELINE MATERNAL CHARACTERISTICS** | | | |
| Maternal age *Mean (SD)* | 31.0 (5.7) | 29.7 (5.5) | 31.6 (5.8) |
| Height  *Mean (SD)* | 163 (15.9) | 156 (6.4) | 166 (6.5) |
| BMI  *Mean (SD)* | 25.5 (6.0) | 21.7 (3.6) | 28.6 (6.7) |
| Birthweight *Median (IQR)* | 2570 (2289.3 – 2790) | 2184 (1924 – 2300) | 2230 (1866 – 2410) |
| Gestation at delivery  *Median (IQR)* | 251 (241 – 255) | 250 (348 – 253) | 249 (235 – 256) |
| Indigenous (%) | 227 (1.4) | 3 (0.8) | 29 (2.5) |
| Nulliparous (%) | 8203 (49.8) | 316 (79.4) | 343 (29.1) |
| Male infant (%) | 8940 (54.3) | 214 (53.8) | 626 (53.1) |
| **OBSTETRIC AND PERINATAL OUTCOMES** | | | |
| **Perinatal mortality**  Total (%)  Stillbirth (%)  Perinatal death (%) | 375 (2.3)  *Ref*  275 (1.7)  *Ref*  100 (0.61)  *Ref* | 51 (2.7)  *1.20 (0.90 – 1.60, p = 0.22)*  46 (2.5)  *1.47 (1.08.- 2.01, p = 0.01)*  5 (0.27)  *0.44 (0.18 – 1.08)* | 85 (3.2)  *1.41 (1.19 – 1.78, p = 0.004)*  77 (2.9)  *1.74 (1.36 – 2.24, p < 0.001)*  8 (0.30)  *0.50 (0.24 – 1.02, p = 0.052)* |
| **Low Apgars**  Ap^5^ <4 (%)  Ap^5^ <7 (%) | 360 (2.2)  *Ref*  1105 (6.7)  *Ref* | 50 (2.7)  *1.22 (0.91 – 1.64, p =0.18)*  145 (7.8)  *1.16 (0.98 – 1.3, p = 0.09)* | 85 (3.2)  *1.47 (1.16 – 1.85, p = 0.001)*  215 (8.1)  *1.21 (1.05 – 1.39)*** |
| **NICU admission (%)** | 1618 (9.8)  *Ref* | 215 (11.5)  *1.17 (1.02 – 1.34, p = 0.02)* | 330 (12.5)  *1.27 (1.14 – 1.42, p < 0.001)* |
| **Suspicion of poor growth**  Operative delivery for fetal distress (%)  Induction or operative birth for suspected poor fetal growth (%) | 1901 (11.5)  *Ref*  342 (2.1)  *Ref* | 328 (17.5)  *1.52 (1.37 – 1.69, p < 0001)*  253 (13.5)  *6.51 (5.58 – 7.61, p < 0.001)* | 412 (15.6)  *1.35 (1.22 – 1.49, p < 0.001)*  329 (12.4)  *5.99 (5.17 – 6.92, p < 0.001)* |
| **Caesarean section rate**  Total (%)  Planned CS (%)  Emergency CS (%) | 6465 (39.3)  *Ref*  1874 (11.4)  *Ref*  4591 (27.9) | 933 (49.9)  *1.27 (1.21-1.34, p < 0.001)*  262 (14.0)  *1.23 (1.09 – 1.3, p < 0.001)*  671 (35.9)  *132 (1.20 – 146, p < 0.001)* | 1338 (50.6)  *1.29 (1.23 – 1.34, p < 0.001)*  398 (15.0)  *1.32 (1.20 – 1.46, p <0.001)*  940 (35.5)  *1.27 (1.20 – 1.35, p < 0.001)* |

Data presented as number (%) and relative risk ratio (95%CI, p-value)

AGA_all_ = >10^th^ centile and <90^th^ centile by all intrauterine charts, SGA_IG_AGA_WHO_ = <10^th^ centile by INTERGROWTH fetal charts but >10^th^ centile by WHO fetal charts, SGA_WHO_AGA_IG_ = <10^th^ centile by WHO fetal charts but >10^th^ centile by INTERGROWTH fetal charts, SGA_IG_AGA_GROW_ = <10^th^ centile by INTERGROWTH fetal charts but >10^th^ centile by GROW customised centiles, SGA_GROW_AGA_IG_ = <10^th^ centile by GROW customised centiles but >10^th^ centile by INTERGROWTH fetal charts.
