# Supplementary material for: Identification of the optimal growth charts for use in a preterm population: An Australian state-wide retrospective cohort study
Source: PLoS Med. 2019 Oct 4;16(10):e1002923. doi: 10.1371/journal.pmed.1002923 (PMC6777749; doi:10.1371/journal.pmed.1002923)
Supplement: S1 Text — (DOCX) [file pmed.1002923.s004.docx]

**ANALYSIS PLAN**

**DATA MANAGEMENT**

Prior to data cleaning and analysis, a plan was formulated to determine the inclusion and exclusion criteria, how gestational age would be defined in the case of stillbirth, and how implausible data values would be managed in the analysis. This plan is outlined below.

**Exclusion criteria:**

- Multiple pregnancies
- Congenital anomalies (of any kind)
- Termination of pregnancy (given primary outcomes of neonatal death and stillbirth could not be measured)
- <24.0 weeks gestation excluded (significant variation in resuscitation preferences and outcomes)
- >42+6 week’s gestation (rare and already known to be associated with adverse outcomes irrespective of birthweight)

**Plan for missing or implausible data:**

Decisions about dealing with missing/implausible data were made based on Australian census data which provided the extremes of maternal characteristics (eg. maternal age) associated with live birth, in addition to information from the perinatal GROW institute which detailed implausible data cut-offs based on their large datasets.

- Those with missing fetal sex were removed as all growth charts were sex specific
- Those with missing birthweights would be removed given the nature of the study
- Ages <13 were considered missing data for maternal age
- Those with missing parity were still included but would not have a completely customised centile
- Weight would be coded as missing if <35kg, and height as </= 125cm, or height and weight excluded if BMI <14.0kg/m2
- Only those with data on gestation by days were included, as day by day growth standards were used in our analysis
- Implausible birthweights were removed if marked as implausible after review of our dataset by the GROW Perinatal institute

**EXPLORATION OF DIFERENT GROWTH CHARTS**

**Decision regarding which growth charts to use:**

We discussed our study objectives (exploring the utility of different growth charts amongst a preterm population) extensively amongst our authorship team, and selected a feasible number of charts to explore using the rationale outlined below.

- Our goal was to focus on growth standards with the greatest international applicability, as well as an Australian population-specific dataset
- Population birthweight centiles were those published by Dobbins et al in 2012 [24], which are the most contemporary Australian population birthweight charts, and considered to be the standard amongst this population
- INTERGROWTH and WHO charts (fetal and birthweight) were used as they were the largest international growth charts and so had the greatest potential international applicability
- GROW charts were used as the most internationally validated customised growth standard
- Initial analysis was planned to look at the proportion classified as SGA by both intrauterine and birthweight standards. We hypothesised that a much greater and more representative proportion would be classified as SGA by intrauterine growth standards. If this was to be confirmed by our dataset then we planned to focus on identifying the optimal fetal growth standard to use in this population.

**Customisation of GROW centiles:**

- Ethnicity was not included as a variable in the analysis due to limitations of the dataset which included data on self-identified country of birth only. Country of birth has been shown to be a poor surrogate for ethnicity in Australia where the population is characterised by high rates of migration and interethnic marriage
- All other factors (fetal sex, parity, maternal height and weight) were included, although we acknowledge that nulliparity, while associated with reduced birthweight, is also associated with increased stillbirth risk. This was included despite this in order to maximise the number of customisable characteristics, as this has been the most widely adopted approach in the published literature.

**INITIAL ANALYSES**

**Gestational age classification of stillbirths:**

- Stillbirths were examined in two ways:
  1. Unadjusted gestational age at birth
  2. Gestational age *adjusted* for the likely time interval between fetal demise and delivery (48 hours prior), in order to avoid overestimating the relative proportions of stillborn infants that were SGA

**Outcomes to be investigated:**

- Primary outcome: stillbirth and combined perinatal mortality (stillbirth or neonatal death within 28 days of delivery)
- Secondary outcomes:
  - Five minute Apgar score of <7 or <4
  - Admission to NICU
  - Additional care received during the antenatal period for poor fetal growth
  - Poor fetal growth as a reason for induction of labour or operative birth
  - Planned and unplanned caesarean section rate

**Initial analysis plan:**

- The SGA population was calculated for each chart to identify relative proportions (<10^th^ centile)
- ‘Non-overlapping’ SGA populations were also identified ie. those classified as SGA by one definition but not by another
- Two additional cohorts/sub-populations were defined:
  - SGA_all_ <10^th^ centile by all intrauterine charts (a “high-risk” population)
  - AGA_all_ >10^th^ and <90^th^ centile by all intrauterine charts (a relatively healthy population)
- Outcomes were compared using ORs between the populations identified by each chart in its entirety and between the sub-populations defined above
- p<0.05 was considered statistically significant
- Statistical analysis was conducted using GraphPad PRISM

**REVIEW OF SUB-POPULATIONS CAPTURED BY EACH CHART**

**A focus on intrauterine growth charts:**

- A much greater proportions of infants were classified as SGA by intrauterine standards, and higher proportions of stillborn infants were also classified as SGA by intrauterine standards
- This was felt to be in keeping with the pathophysiology of preterm birth, we decided to focus on comparisons between intrauterine growth standards

**Comparisons between intrauterine growth standards:**

- Initially, primary comparisons were planned between non-overlapping populations classified by each chart
- However, the population classified as SGA only by INTERGROWTH fetal charts was negligible compared to WHO and GROW charts, and so this was not a feasible cohort for comparison
- Instead, comparisons between non-overlapping populations were compared between WHO and GROW charts only
- The analysis focussed on WHO and GROW non-overlapping populations as they were the charts that identified the most similarly sized populations of SGA infants

**FINAL ANALYSES (following revisions requested by PLOS Medicine Editors/Reviewers)**

**Further statistical analysis:**

- After review by the PLOS Medicine statistician and discussion with our authorship team and local statistician, our statistical approach was amended
- Outcomes were reported as point estimates with Wilson 95% confidence intervals, both overall and for each gestational age-based strata
- Relative risks, as opposed to odds ratios, were used given they were more easily understandable in a clinical context and our study included an entire state population cohort
- Re-classification of outcomes due to application of the five SGA growth standards determined that the outcome proportions were clustered within infants and this must be accounted for in the subsequent analysis
  - Fixed effect conditional Poisson regression with robust variance estimate was used to provide relative risk ratios, 95% confidence intervals and p-values between different growth charts
  - Gestational age-based strata were incorporated into the regression model
  - Significance level was two-sided, set at 0.05 and not adjusted for multiple comparisons
- Initially, statistical analysis was planned between SGA cohorts in their entirety (eg. SGA by INTERGROWTH or SGA by WHO charts), however this was re-considered after formal statistical review given the overlap between these populations, and as a result, this was not included in the final analysis
- Final statistical analyses were conducted using Stata Version 14
